# The neuro-pianist

**DOI:** 10.3389/fnsys.2013.00035

**Published:** 2013-07-31

**Authors:** Eitan Globerson, Israel Nelken

**Affiliations:** ^1^Gonda Multidisciplinary Brain Research Center, The Jerusalem Academy of Music and Dance, Bar Ilan UniversityJerusalem, Israel; ^2^Department of Neurobiology, The Edmond and Lily Safra Center for Brain Sciences, Silberman Institute of Life Sciences, Hebrew UniversityJerusalem, Israel

## Introduction

Music performance is considered one of the most complex human activities. It involves not only hundreds of muscles, coordinated to produce the desired musical result, but also a variety of cognitive mechanisms, including complex emotional and analytic processes (Zatorre et al., 2007). The study of music performance has yielded important insights into brain processes, including neural plasticity (Schlaug et al., 1995; Schlaug, 2001; Münte et al., 2002; Schneider et al., 2005), motor control (Slobounov et al., 2002; Watson, 2006), rhythmic control (Rammsayer and Altenmüller, 2006; Repp and Doggett, 2007; Goebl and Palmer, 2009), and emotional communication (Gabrielsson and Juslin, 1996; Juslin, 1997; Juslin and Laukka, 2003). The information acquired through systematic studies is invaluable in its contribution to our understanding of brain mechanisms underlying music perception and performance. However, such studies are limited in their ability to simulate the atmosphere of a concert performance, or to systematically follow the long period of training required to master a musical piece. Hence, it may be beneficial to obtain additional information by studying the strategies employed by professional concert artists to optimize their practice routines and their performance under stressful conditions. Such strategies enable them to confront many of the physiological constraints dictated by the muscular and central nervous system. In this short note, we highlight some key properties of these strategies and their possible relevance to studying other complex human activities

## Distributing control over time scales

Performing virtuoso musical pieces often involves extremely fast motor action. In piano playing, for example, a pianist may play at a speed of 30 notes per second (Rumelhart and Norman, 1982), surpassing visual reaction times (Lashley, 1951). Many other human activities, such as speech, competitive gymnastics, driving a car, typing, as well as many other activities involve extremely fast serial motor actions. Lashley, in a seminal article published in 1951, observed that the time differences between the components of fast sequential action do not allow separate conscious planning of each component He concluded that the ability to perform fast sequential motor actions can be explained only by the existence of a single motor plan encompassing the whole sequence. This hierarchical movement planning theory is also supported by evidence showing that the process of learning complex serial movements typically involves formation of “chunks” of movements (Miller, 1956). The process of learning a task is habitually associated with a reduction in movement variability and a decreasing involvement of cognitive control (Cohen and Poldrack, 2008). This state, commonly termed *automaticity*, enables an individual to perform a task without reduction in performance in the presence of a concurrent competing task (Logan, 1979).

The time scales involved in music performance suggest that both automatic and non-automatic processes are involved. While fast passages may involve time scales of tens of milliseconds (Rumelhart and Norman, 1982), musical phrases, sections, and whole movements typically involve time scales of seconds, minutes, and longer. In performing such musical excerpts, the performer may consciously follow various structural levels of the piece using known nomenclature such as “exposition,” “first theme,” or “second theme,” etc. Hence, musicians must rely on both implicit and explicit memory processes during music performance, enabling them to play fast passages, relying on automaticity, while simultaneously dedicating their intellectual and emotional resources to higher level processes.

Concert performances may involve high levels of stress. If musicians begin to doubt their knowledge of a musical text, they may consequently doubt their ability to automatically execute sequential motor action required to play the same musical text. Such hesitation may drive the performer to use alternative motor planning strategies, namely, on-line movement-by-movement planning. Since fast passages cannot be executed using on-line motor planning, the ultimate outcome of this strategy may be a failure to perform the musical passage in a successful manner, leading to growing anxiety, which, in turn, increases the uncertainty in both explicit and implicit memory abilities. The final outcome of this Performance Vicious Cycle (PVC) is a faulty performance, which, at times, may even reach full interruption of the musical performance.

Diverse strategies are employed by musicians to overcome such incidents. Those habitually comprise cognitive behavioral treatment (Kendrick et al., 1982; Harris, 1987; Nagel et al., 1989), various relaxation techniques (Sweeney and Horan, 1982; Niemann et al., 1993), pharmacological methods, such as beta blockers which reduce sympathetic activation by stress (Neftel et al., 1982; Nube and Musicobgy, 1991), or even hypnosis (Stanton, 1994). The efficacy of all these methods is debatable (Kenny, 2005). The reason for this may be that most of the reported interventions attempt to affect the general state of mind of the performing musician, but do not try to directly affect mental processes during the performance of specific pieces of music.

A more direct approach for a performer to avoid the PVC is by developing “mental scripts” which include the exact series of desirable mental events during a musical performance. These scripts should not include any reference to fingering or other fast-scale motor action, but rather focus on large time-scale events, such as musical phrases. Thus, the performer can actively avoid interfering with automaticity. These mental scripts should be continuously repeated, in order to acquire automatic control of the desired mental process, in a similar fashion to the music practice routines, which use constant repetition to produce faultless and automatic motor control. By employing this method, musicians may reach a relatively high state of certainty in their ability to maintain their technical and mental achievements, earned through long years of practicing.

The same approach may potentially apply to other forms of complex sequential activities, such as sports, dance, and even speech. The reason why certain competitive gymnasts succeed more than others in producing perfect drills is not necessarily only due to physiological superiority, but also to the employment of mental scripts which do not allow the PVC to begin. This line of thought can be tested in studies examining other forms of fast sequential motor action. Mental scripts fitting specific tasks can be developed, rehearsed and then tested under stressful conditions to examine the ability of the subject to maintain automaticity. Such mental scripts should involve deliberate thinking in relatively slow time scales. The scripts could be related to the task (for example, naming the finger number, simultaneously with the beginning of a finger-tapping sequence), or completely unrelated to the task (reading or mentally rehearsing a certain text). Next, the ability of the subjects to maintain rapid and accurate sequence tapping can be examined, with and without the employment of mental scripts.

## Mental control of bimanual coordination

The ability of humans and primates to coordinate the movements of both limbs has been the focus of scientific research for decades. Several general conclusions regarding the nature of bimanual movements have been established and replicated in various empirical studies. One of the most common observations is that spontaneous bimanual hand movements tend to be similar, spatially (with a preference for mirror movements) and temporally (phase-locked) (Kelso et al., 1979; Kelso, 1984; Franz, 1997) Furthermore, symmetric movements are more natural than parallel movements, which are, in turn, easier to perform than unrelated movements. Corresponding to the behavioral observations, it has been found that brain activation for parallel movements is greater than for symmetric movements (Sadato et al., 1997; Stephan et al., 1999). These findings may suggest that parallel movements are more computationally demanding than symmetric movements.

Interestingly, professional pianists show different activation patterns than naïve individuals for bimanual movements. Complex bimanual finger movements in professional pianists result in less brain activation than in naïve individuals (Haslinger et al., 2004). Moreover, brain activation for parallel movements is not larger than for symmetric movements in pianists. These functional differences could have structural underpinnings. Indeed, differences between musicians and non-musicians were found in brain structures associated with bimanual movement. Increased corpus callosum volume (Schlaug et al., 1995) and reduced trans-callosal inhibition (Ridding et al., 2000) were shown in professional musicians. Since inter-hemispheric connections were found to be highly important for bimanual movement (Serrien et al., 2001; Johansen-Berg et al., 2007; Muetzel et al., 2008), increased inter-hemispheric connectivity could account for higher efficiency in certain bimanual movements in musicians.

**Figure 1:**
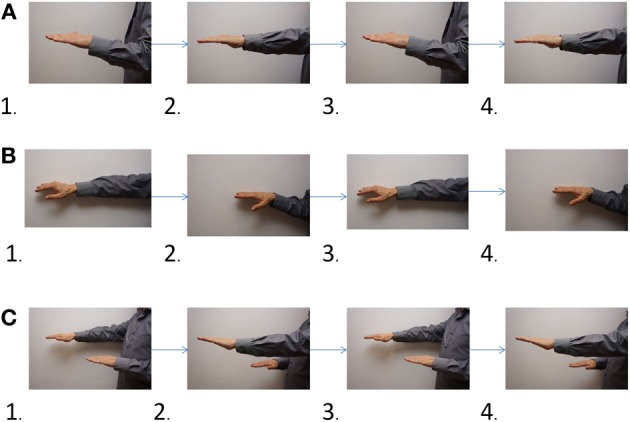
**Demonstrating separate and unified bimanual movement mental coding**. Instructions: first practice each hand alone: **(A)** left hand movement **(B)** right hand movement. Now, try to combine the movements of both hands you rehearsed in stages **(A)** and **(B)**. Now try to imitate the movements shown in section **(C)**, which demonstrates the resulting bimanual movement. You would probably find it easier to start directly with bimanual movement, than to try to combine the movements of each hand which were rehearsed separately.

However, the unique brain activation patterns in pianists could be also attributed to different strategies employed by musicians to cope with non-symmetric bimanual movements. When practicing bimanual passages, pianists have to choose between mastering each hand alone first, or, alternatively, practicing both hands together from day one. In choosing between these two strategies, pianists employ an intuitive knowledge that certain bimanual movements are easier to execute as a unit, while other movements may involve two discrete motor plans which are combined later to produce coordinated movement. To illustrate how effective this can be, let us perform the following simple experiment: position your left hand close to the body, and then move it forwards, backwards, forwards and finally backwards again. Now, position your right arm in a forward position (away from the body), and move it backwards (toward the body) then forward (away from the body) then backwards and finally forward again. Now, try to perform the movements you rehearsed for each hand simultaneously (i.e., both hands together). You will probably find this drill uncomfortable to perform at first. Now, let's try a different approach: hold your hands in the following starting position: the right hand away from the body, and the left hand close to the body. Now move your hands in a rowing movement, so that the hands perform symmetrical movements (it is also possible to think of this movement as a “karate” movement). You will probably find this strategy easier and faster to accomplish (see Figure 1 for illustrated instructions).

In choosing between a unified-bimanual motor scheme and two independent learning processes for each hand, pianists unconsciously enter the years-long discussion confronting two different theories of bimanual movement. The first theory suggests a “generalized motor program.” This theory was originally proposed by Schmidt (1975), who suggested that bimanual movement is governed by a unifying motor plan (a “generalized motor program”), rather than a combination of discrete plans for each component of the movement (Schmidt, 1975). The second theory, proposed by Marteniuk et al. (1984), suggests that bimanual movements are governed by two independent motor plans for each limb (“inter-hemispheric cross talk”), unified to produce a common movement (Marteniuk et al., 1984).

Single-neuron recordings in macaque motor cortex (Donchin et al., 1998) showed that neurons in each hemisphere control the motion of both limbs. Later work of the same group (Rokni et al., 2003) suggested that inhibitory cross-callosal effects act to decorrelate the unimanual and bimanual representations, suggesting that bimanual representations are distinct from the representations of the two unimanual components. While both of these results suggest the existence of a single, bimanual motor plan, it is beyond the scope of the present paper to discuss each of these theories and its advantages and shortcomings.

The experience of piano playing may suggest that multiple coding exist for the same bimanual movements. Hence, pianists may choose the optimal coding for bimanual movement using mental control during practice. This proposition is relatively simple to check in controlled conditions, by directing participants to switch between different mental representations of bimanual movement, and examining the resulting motor performance.

## Conclusion

In this short paper, two highly complex human actions, typical of music performance, were discussed. We suggest that in both fast sequential action and complex bimanual control, it is possible to dramatically improve performance by employing ready-made mental scripts. Hence, music performance can serve as an archetypical paradigm for studying deliberate mental control of complex motor action. Experimental paradigms involving complex motor action usually focus on the actual behavioral results, but less on the state of mind and thoughts of the participants during execution of the tasks. By designing mental scripts and incorporating them in controlled experiments, it may be possible to demonstrate a significant effect on motor performance and on the concomitant brain activity. Such experiments may deeply change our understanding of an array of complex and challenging human activities.
